# Evaluation of *in vitro* efficacy of aztreonam-nacubactam and cefepime-nacubactam against clinical isolates of *Stenotrophomonas maltophilia*

**DOI:** 10.1128/aac.00755-25

**Published:** 2025-09-22

**Authors:** Wataru Aoki, Yoshifumi Uwamino, Hiroaki Kubota, Yuka Kamoshita, Rika Inose, Mika Nagata, Ho Namkoong, Hiromichi Matsushita

**Affiliations:** 1 Clinical Laboratory, Keio University Hospital 592832, Tokyo, Japan; 2 Department of Laboratory Medicine, Keio University School of Medicine 12869, Tokyo, Japan; 3 Department of Infectious Diseases, Keio University School of Medicine 12869, Tokyo, Japan; 4 Department of Microbiology, Tokyo Metropolitan Institute of Public Health, Tokyo, Japan; University of Fribourg, Fribourg, Switzerland

**Keywords:** nacubactam, *Stenotrophomonas maltophilia*

## Abstract

We evaluated the *in vitro* activity of aztreonam-nacubactam (ATM-NAC) and cefepime-nacubactam (FEP-NAC) against 53 clinical isolates of *Stenotrophomonas maltophilia* from blood cultures. Minimum inhibitory concentrations (MICs) were determined via broth microdilution. While ATM-NAC showed more significant MIC reduction, FEP-NAC also remarkably decreased MICs, despite cefepime being a substrate for L1 β-lactamase. These findings suggest the potential of β-lactam–NAC combinations as promising alternatives, warranting further *in vivo* studies to confirm clinical applicability.

## INTRODUCTION

Diazabicyclooctanes (DBOs) are non-β-lactam β-lactamase inhibitors that can inhibit class A and C β-lactamases, and some class D enzymes (1). Recently, DBOs such as avibactam (AVI) and relebactam have been combined with β-lactam antibiotics (e.g., ceftazidime and imipenem) to treat infections caused by drug-resistant gram-negative bacteria. However, a known limitation of DBOs is their inability to inhibit metallo-β-lactamases (MBLs), classified as class B enzymes.

Newly developed DBOs such as nacubactam (NAC, RG6080/OP0595; Roche, Fedora, Meiji) and zidebactam (ZID, WCK5107; Wockhardt) have demonstrated high affinity for penicillin-binding protein 2 (PBP2) in gram-negative bacilli, showing intrinsic antibacterial activity. When combined with β-lactam antibiotics that target PBP3, these agents exert an “enhancer effect,” improving activity even against MBL-producing organisms (2, 3).

*Stenotrophomonas maltophilia* harbors both L1 β-lactamase (an MBL) and L2 β-lactamase (class A), which confer resistance to most conventional β-lactams, including carbapenems. Historically, treatment options have been limited to trimethoprim-sulfamethoxazole, levofloxacin, or tetracyclines. More recently, cefiderocol (FDC), a siderophore cephalosporin with demonstrated activity against *S. maltophilia*, has become available (4). Additionally, aztreonam (ATM)—a monobactam not hydrolyzed by MBLs—combined with AVI has emerged as a promising therapeutic option. This combination is recommended in the Infectious Diseases Society of America guidelines (5).

We previously reported that the ATM-AVI combination and FDC showed strong *in vitro* activity against *S. maltophilia* blood culture isolates from a university hospital in Tokyo (6, 7). Although AVI does not enhance ATM’s activity via PBP binding, NAC may potentiate ATM’s effect through PBP2 binding and an enhancer mechanism. Similarly, combinations of cefepime (FEP) with ZID have demonstrated activity despite FEP being a substrate for the L1 enzyme (8). However, no study to date has evaluated the *in vitro* activity of ATM or FEP in combination with NAC against *S. maltophilia*.

In this study, we assessed the *in vitro* activity of ATM-NAC and FEP-NAC against 53 previously collected blood culture isolates of *S. maltophilia* from Keio University Hospital (Tokyo, Japan) between January 2012 and December 2024. Patient background and identification methods were previously described (6). Whole-genome sequencing data from our prior study (7) were used to determine sequence types using MLST 2.0 (9), assess clonality with Snippy v3.2 (https://github.com/tseemann/snippy) and Gubbins v2.3.4 (10), and detect *bla*_L1_ and *bla*_L2_ genes by BLAST v2.12.0+ with the BLASTN algorithm (11), referencing EF126059 and EF126086, respectively.

Minimum inhibitory concentrations (MICs) were determined by broth microdilution using CAMHB in accordance with CLSI M100 35th edition guidelines (12), over a concentration range of 0.063–128 µg/mL. NAC was combined with ATM or FEP at a 1:1 ratio per CLSI guidance. Quality control for NAC used *Escherichia coli* ATCC 25922 and *Pseudomonas aeruginosa* ATCC 27853; other agents were tested with *Klebsiella pneumoniae* ATCC 700603.

MIC_50_ and MIC_90_ values were calculated for each agent and combination. Statistical analysis of MIC differences (with/without NAC) was performed using the Wilcoxon signed-rank test after log transformation. Statistical analyses were conducted using JMP Student Edition 18. MICs > 128 µg/mL were treated as 256 µg/mL for statistical purposes.

The isolates showed diverse sequence types and were polyclonal in phylogenetic analysis. All but three strains harbored both *bla*_L1_ and *bla*_L2_ (Fig. S1; Table S1). MIC_50_ and MIC_90_ for NAC and ATM alone were both >128 µg/mL, while for FEP alone were 32 and 64 µg/mL, respectively.

**Fig 1:**
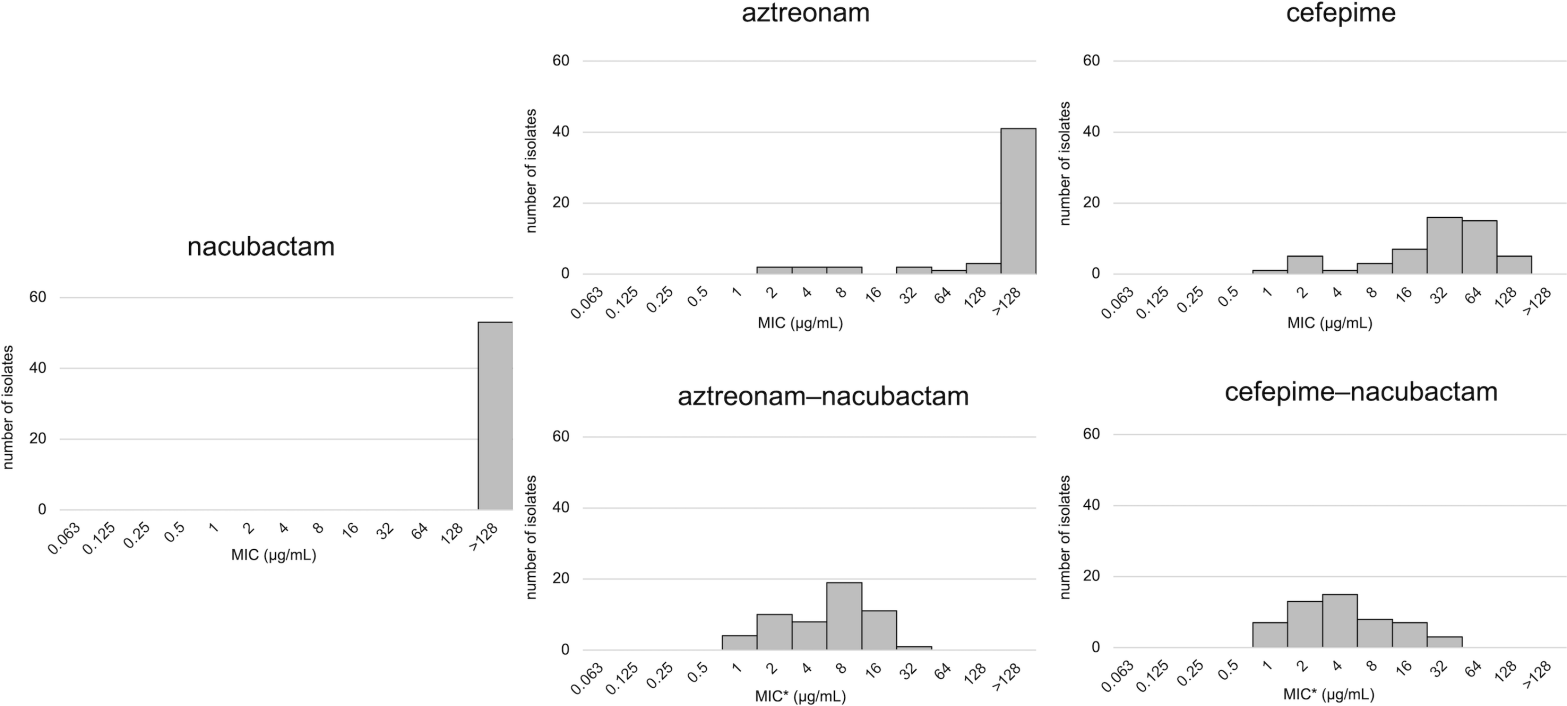
Distribution of MIC values for aztreonam, cefepime, nacubactam alone, and their respective combinations. The distribution of minimum inhibitory concentrations (MICs) for aztreonam, cefepime, and nacubactam as monotherapies, as well as for the combinations of aztreonam–nacubactam and cefepime–nacubactam, is demonstrated. *For the β-lactam and nacubactam combinations, the two agents were used at a 1:1 ratio, and the MIC values are represented based on the β-lactam concentration.

**Fig 2:**
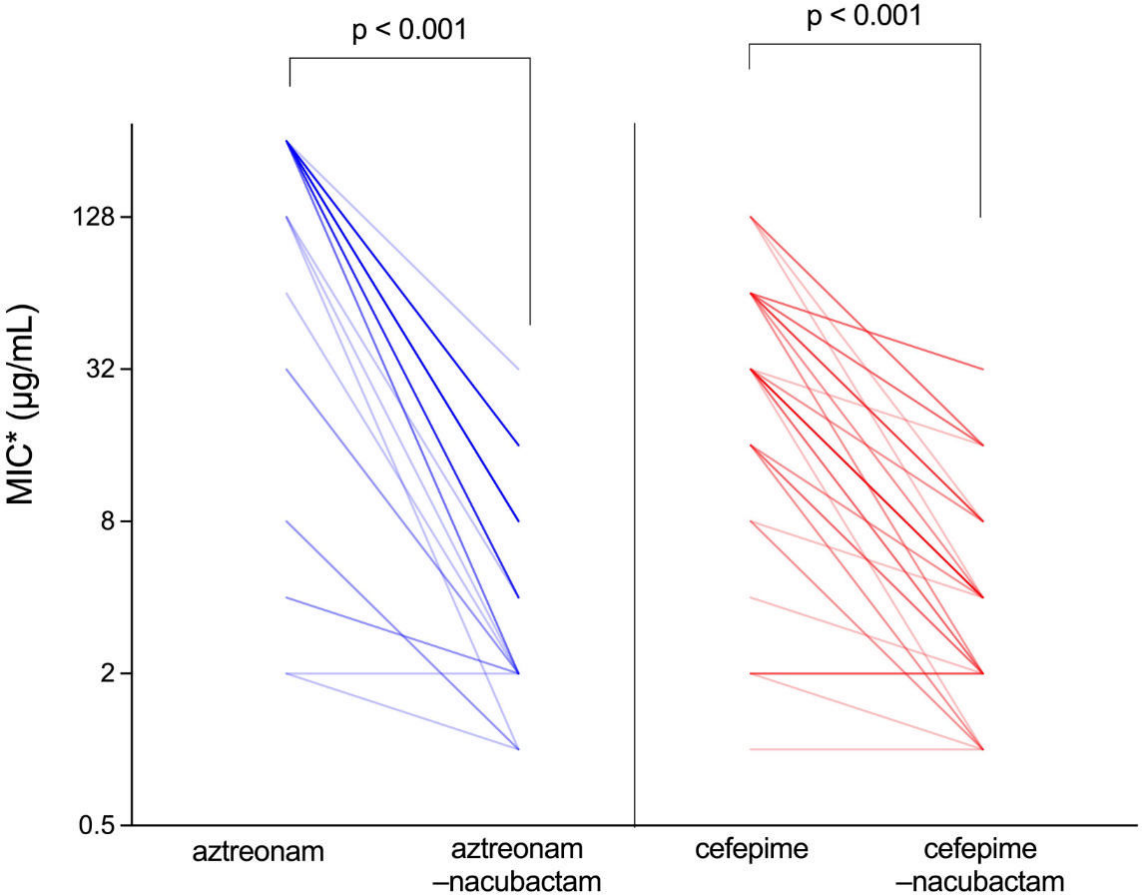
The effect of nacubactam combination on MIC reduction. The *in vitro* reduction in minimum inhibitory concentrations (MICs) of aztreonam and cefepime after the addition of an equivalent amount of nacubactam is demonstrated. The thickness of the lines corresponds to the number of overlapping isolates.

With NAC, MIC_50_/MIC_90_ values were reduced to 8/16 µg/mL for ATM-NAC and 4/16 µg/mL for FEP-NAC. ATM-NAC showed slightly higher MICs than FEP-NAC (*P* = 0.018) (Fig. 1). Both combinations significantly reduced MICs compared to monotherapy (*P* < 0.001) (Fig. 2). Median fold reduction was 32-fold (interquartile range [IQR]: 16–32) for ATM and 8-fold (IQR: 4–8) for FEP, with greater reduction for ATM (*P* < 0.001).

To our knowledge, this is the first study assessing ATM-NAC and FEP-NAC against *S. maltophilia*. Although ATM-NAC showed MIC reductions, MIC_50_/MIC_90_ values for ATM-AVI were slightly lower than those for ATM-NAC. FDC demonstrated even lower MIC_50_/MIC_90_ values (Table S2). Interestingly, despite FEP being a known substrate of L1 β-lactamase, FEP-NAC also showed significant MIC reductions, suggesting that NAC may confer an enhancer effect through PBP2 targeting.

Previous studies, such as that by Sadar et al., reported MIC_50_/MIC_90_ values of 4/16 µg/mL for the FEP-ZID combination against *S. maltophilia* (8), indicating that FEP-NAC and FEP-ZID may have comparable activity. Another relevant combination is FEP-taniborbactam (FEP-TAN). Taniborbactam is a cyclic boronic acid β-lactamase inhibitor with broad-spectrum inhibitory activity against class A, C, and D β-lactamases, as well as subclass B1 MBLs such as NDM and VIM. However, it does not inhibit subclass B3 MBLs, including L1, which is characteristic of *S. maltophilia* (13, 14). Therefore, the *in vitro* activity of FEP-TAN against *S. maltophilia* is thought to derive primarily from inhibition of the L2 β-lactamase (15). According to the report by Gerges et al., the MIC_50_/MIC_90_ values of *S. maltophilia* isolates recovered from blood cultures of cancer patients for FEP-TAN were 4/8 µg/mL (16). Although not a direct comparison, FEP-NAC demonstrated relatively comparable *in vitro* activity to FEP-TAN.

Although there is no established breakpoint for FEP-NAC against *S. maltophilia*, CLSI has proposed a provisional susceptibility breakpoint of 8 µg/mL for Enterobacterales. Based on this threshold, 81.1% of our isolates would be considered susceptible.

Given that ATM and FDC are WHO AWaRe “RESERVE” category antibiotics (https://aware.essentialmeds.org/list), preserving their use is desirable. Developing alternative options using WATCH category agents like FEP, especially in combination with novel DBOs such as NAC, could be valuable. However, as NAC is not yet approved for clinical use, further pharmacokinetic/pharmacodynamic and *in vivo* efficacy studies are warranted.

In conclusion, both ATM-NAC and FEP-NAC combinations demonstrated promising *in vitro* activity against clinical blood isolates of *S. maltophilia*, suggesting their potential as alternative therapeutic options pending further clinical development.

## SUPPLEMENTAL MATERIAL

Supplemental material aac.00755-25-s0001.pdf Fig. S1; Tables S1 and S2.

ASM does not own the copyrights to Supplemental Material that may be linked to, or accessed through, an article. The authors have granted ASM a non-exclusive, world-wide license to publish the Supplemental Material files. Please contact the corresponding author directly for reuse.
