# Supplementary material for: Evaluation of in vitro efficacy of aztreonam-nacubactam and cefepime-nacubactam against clinical isolates of Stenotrophomonas maltophilia
Source: Antimicrob Agents Chemother. 2025 Sep 22;69(11):e00755-25. doi: 10.1128/aac.00755-25 (PMC12587625; doi:10.1128/aac.00755-25)
Supplement: Supplemental material — Fig. S1; Tables S1 and S2. [file aac.00755-25-s0001.pdf]

Supplementally files

Table S1. Sequence types and presence of *bla*L1 and *bla*L2 genes in the isolates

Isolate number	Sequence type	*bla*L1	*bla*L2
S1	unidentified	✓	✓
S2	28	✓	✓
S3	621	✓	✓
S4	unidentified		
S5	714	✓	✓
S6	77	✓	✓
S7	unidentified	✓	✓
S8	77	✓	✓
S9	28	✓	✓
S10	94	✓	✓
S11	94	✓	✓
S12	151	✓	✓
S13	unidentified	✓	✓
S14	unidentified	✓	✓
S15	unidentified	✓	✓
S16	621	✓	✓
S17	unidentified	✓	✓
S18	unidentified		
S19	904	✓	✓
S20	77	✓	✓
S21	unidentified	✓	✓
S22	496	✓	✓
S23	77	✓	✓
S24	unidentified	✓	✓
S25	27	✓	✓
S26	162	✓	✓
S27	94	✓	✓
S28	unidentified	✓	✓
S29	28	✓	✓
S30	77	✓	✓
S31	unidentified	✓	✓
S32	208	✓	✓
S33	27	✓	✓
S34	unidentified	✓	✓
S35	828	✓	✓
S36	120	✓	✓
S37	unidentified		
S38	31	✓	✓
S39	unidentified	✓	✓
S40	unidentified	✓	✓
S41	886	✓	✓
S42	31	✓	✓
S43	94	✓	✓
S44	4	✓	✓
S45	621	✓	✓
S46	24	✓	✓
S47	210	✓	✓
S48	212	✓	✓
S49	unidentified	✓	✓
S50	27	✓	✓
S51	unidentified	✓	✓
S52	unidentified	✓	✓
S53	unidentified	✓	✓

* Sequence types were determined using MLST 2.0[1] based on whole-genome sequencing data. Isolates for which the sequence type could not be identified are labeled as "unidentified."

** The presence of blaL1 and blaL2 was assessed by BLASTN analysis[2] using default parameters, with EF126059 (blaL1) and EF126086 (blaL2) from the NCBI Reference Gene Catalog (https://www.ncbi.nlm.nih.gov/pathogens/refgene/) as the subject reference sequences. Genes were considered present when a significant hit was detected.

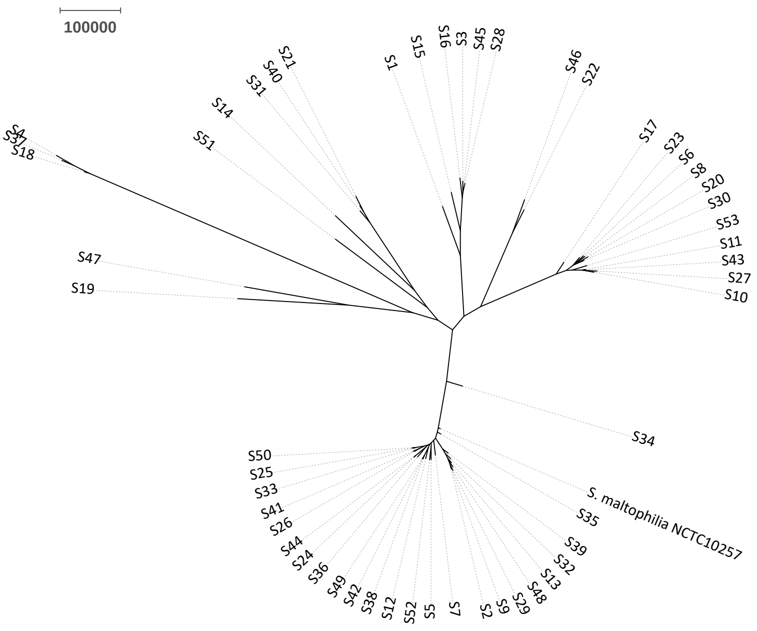

Figure S1. Phylogenetic tree of isolates based on core genome SNP analysis. Whole-genome assemblies were mapped to the *S. maltophilia* NCTC10257 reference genome (NZ_LT906480) using Snippy v3.2 (https://github.com/tseemann/snippy) to generate whole-genome alignment. Core genome SNPs located outside of putative recombinant regions were used to construct a phylogenetic tree with Gubbins v2.3.4[3], applying a filter percentage of 50%. The scale bar represents the number of nucleotide substitutions.

Table S2. MIC distributions of ATM-NAC and FEP-NAC among the isolates, in comparison with those of ATM-AVI and FDC

**MIC**		**≤0.06**	**0.12**	**0.25**	**0.5**	**1**	**2**	**4**	**8**	**16**	**32**	**64**	**128**	**>128**	**MIC_{50} (µg/mL)**	**MIC_{90} (µg/mL)**
ATM-NAC		0	0	0	0	4	10	8	19	11	1	0	0	0	8	16
FEP-NAC		0	0	0	0	7	13	15	8	7	3	0	0	0	4	16
ATM-AVI		0	0	0	0	2	23	22	5	1	0	0	0	0	4	8
**MIC**	**≤0.03**	**0.06**	**0.12**	**0.25**	**0.5**	**1**	**2**	**4**	**8**	**16**	**32**	**>32**			**MIC_{50} (µg/mL)**	**MIC_{90} (µg/mL)**
FDC	19	12	9	8	3	1	1	0	0	0	0	0			0.06	0.25

MIC distributions of 53 isolates used in this study are shown. Data for ATM-AVI and FDC were cited from our previously published studies[4,5].
Abbreviations: ATM, aztreonam; NAC, nacubactam; FEP, cefepime; AVI, avibactam; FDC, cefiderocol

References in supplementary files

1. Larsen MV, Cosentino S, Rasmussen S, Frils C, Hasman H, Marvig RL, Jelsbak L, Sicheritz-Pontén T, Ussery DW, Aarestrup FM, Lund O. 2012. Multilocus sequence typing of total-genome-sequenced bacteria. J Clin Microbiol 50:1355-1361.

2. Camacho C, Coulouris G, Avagyan V, Ma N, Papadopoulos J, Bealer K, Madden TL/ 2009. BLAST+: architecture and applications. BMC Bioinformatics 10:421.

3. Croucher NJ, Page AJ, Connor TR, Delaney AJ, Keane JA, Bentley SD, Parkhill J, Harris SR. 2015. Rapid phylogenetic analysis of large samples of recombinant bacterial whole genome sequences using Gubbins. Nucleic Acids Res 43:e15.

4. Aoki W, Uwamino Y, Kamoshita Y, Inose R, Nagata M, Hasegawa N, Matsushita H. 2025. In vitro activity of aztreonam-avibactam combination against blood culture isolates of Stenotrophomonas maltophilia in Japan before the launch of ceftazidime-avibactam. Microbiol Spectr. 13:e0331624.

5. Aoki W, Uwamino Y, Niida N, Kubota H, Kamoshita Y, Inose R, Nagata M, Ishihara O, Uno S, Yoshifuji A, Namkoong H, Hasegawa N, Matsushita H. 2025. Cefiderocol susceptibility of Stenotrophomonas maltophilia species complex and carbapenem-resistant Pseudomonas aeruginosa isolates from blood cultures at a university hospital in Tokyo, Japan. J Glob Antimicrob Resist 44:251-255.
